# University Day

**Published:** 1901-05

**Authors:** 


**A Monthly Review of Medicine and Surgery.**

*EDITOR:*

WILLIAM WARREN POTTER, M. D.

All communications, whether of a literary or business nature, books for review and exchanges should be addressed to the editor: 284 FRANKLIN STREET, BUFFALO, N. Y.

VOL. XL.—LVI. MAY, 1901. NO. 10.

### UNIVERSITY DAY.

COMMENCEMENT OF MEDICAL AND PHARMACAL DEPARTMENTS.

THE fifty-fifth annual commencement of the Medical Department and the fourteenth of the Department of Pharmacy of the University of Buffalo, were held on Friday, April 26, 1901, at the Teck Theatre, at 11 o'clock a. m. This was a departure from the usual custom, it being the first time in the history of the college when these ceremonies were not held in the evening.

THE MORNING.

The curators held their examinations on the day previous, which left commencement day a clear field to the graduation exercises and the alumni meetings.

Beginning at 9.30 in the college building the following class reunions were held: '51 in faculty room—Thomas D. Strong, President, Westfield; '61 in library—William B. Mann, President, Evanston, Ill.; '71 in lecture hall—Devillo W. Harrington, President, Buffalo; '81 in amphitheatre—Carleton C. Frederick, President, Buffalo; '91 in alumni hall—Edward J. Gilray, President, Buffalo.

These several classes talked over the old college days, polished the links in the chain of affection that binds the alumni to their alma mater and to each other, and then took luncheon together at the Iroquois at 12.30 p. m.

While these interesting scenes were occurring at the college the new graduates—the class of 1901—were receiving their degrees at the Teck Theatre, where a large audience had gathered to witness the ceremonies. After prayer by the Rev. Israel Aaron, of Temple

Beth Zion, the secretary of the faculty, Dr. John Parmenter, announced the following-named candidates for the degree of doctor of medicine:

Class in medicine—Charles E. Abbott, Ph. G., William Armstrong, Charles W. Banta, William Brady, Wilhelm Brauns, Charles V. Brooks, Herbert M. Burritt, William Wallace Carleton, George Henry Davis, Patrick M. Donovan, Arthur Eisbein, John Benedict Frisbee, William Tweedy Getman, Charles D. Graney, W. Stuart Grimes, George McKenzie Hall, J. Ralph Harris, John Albert Hobbie, A.B., Joel Samuels Hooper, Harry Horner Hubbell, James Lyman Hutchinson, Bergen Fred. Illston, John Francis Kane, Alfred Crosgrove Kingsley, Theodore M. Leonard, Paul Otto Luedeke, Frederick Williams Parsons, William R. Paterson, Helena B. Pierson, Ph.B., John William Riley, George W. Schaefer, Edward L. A. Schwabe, Ph.G., M. Elizabeth Schugens, Thomas Edwin Spalding, Oscar W. Steinlein, Roy Gilbert Strong, Isidor R. Tillman, Carl Schurz Tompkins, Ira Putnam Trevett, Harry Radley Trick, Eli H. Vail, John A. Weidman, Lee Adrian Whitney, Roy Henry Wixson, Alfred B. Wright, Frederick Zingsheim.

The officers of the class are: President, Ira Putnam Trevett; vice-president, Roy Gilbert Strong; secretary, John William Riley; treasurer, William Wallace Carleton; marshal, Oscar W. Steinlein.

The Chancellor, the Hon. James O. Putnam, clad in his university gown and cap, then conferred the degree according to the usual formula and in token of which he handed each candidate his diploma, who were called upon the stage in groups. After the administration of the Hippocratic oath, by the dean, Professor Mann, the honors for standing were announced, each of the following-named having been marked above 90: J. B. Frisbie, H. H. Hubbell, W. R. Patterson and Ira P. Trevett.

The candidates for the degree in pharmacy were then presented to the chancellor by Dr. John R. Gray, secretary of the faculty of pharmacy. Their names are as follows: William G. Achilles, William G. Barker, Frank W. Barnum, Oscar F. Beck, Clarence H. Bierman, Abraham J. Boulet, Frank O. Brickman, Frank A. Chase, Benjamin C. Cofeld, Henry P. Davies, Jr., Earle J. DeGolier, George J. Dittly, Jr., LaVerne Doremus, Harley E. Dowman, Michael J. FitzMorris, Charles N. Harlowe, William T. Hickelton, Frank L. Horton, Noyes G. Husk, Rudolph D. Janke, Franklin J. Jones, Merton D. Linger, Mrs. Ella J. Lock, James H. McAdam, William P. McNulty, Daniel A. Miller, Rudolph C. Miller, Ralph B.

Nicholson, Arthur H. Reimann, Lloyd R. Richards, Frederick G. Ritter, Harry L. Rider, Samuel Ruckel, George I. Serrins, Leo W. Stall, George Stoll, Luther A. Thomas, Henry S. Vaughan, and Leland J. Waldock.

Willet H. Mosher received a degree as doctor in pharmacy in the post-graduate course. The Peabody prize of $50 for the highest standing in the pharmacy class was awarded to Rudolph C. Miller, of Buffalo. W. P. McBetty won the $25 prize for the best standing in the junior class.

The doctorate address was then delivered by Dr. W. E. Ford, of Utica, professor of electro-therapeutics in the University, who chose for his subject, "Instruments of Precision." It was a scholarly presentation of the theme and was well received. The benediction followed and thus closed the commencement exercises of 1901. The orchestra played several appropriate airs during the proceedings.

### THE AFTERNOON AND EVENING.

The Alumni Association held its twenty-sixth annual meeting in Alumni Hall, at the University Building, which was called to order at 2.30 o'clock by the president of the Association, Dr. D. W. Harrington, '71, of Buffalo.

After reading the minutes of the previous sessions, receiving reports of the officers and committees and the transaction of miscellaneous business, the election of officers for the ensuing year was proceeded with, resulting as follows: President, Frank H. Moyer, '72, Moscow, N.Y.; first vice-president, A. W. Bayliss, '89, Buffalo; second vice-president, A. W. Henchell, '89 Rochester; third vice-president, Fridolin Thoma, '87 Buffalo; fourth vice-president, Henry T. Benham, '90, Honeoye Falls, N.Y.; fifth vice-president, Jane W. Carroll, '91, Buffalo; secretary, T. H. McKee, '98, Buffalo; treasurer, Herman K. de Groat, '97, Buffalo; trustee for five years, D. W. Harrington, '71, Buffalo; executive committee, A. T. Lytle, '93, chairman; Grover W. Wende, '89; Franklin W. Barrows, '93; Frank H. Moyer, and John Parmenter, *ex-officiis*.

Then came the president's address, which was a short but interesting paper by the retiring president, Dr. D. W. Harrington, and which was well received.

Professor A. M. Phelps, of New York, ex-president of the Medical Society of the State of New York and professor of orthopedic surgery in the New York Post-Graduate Medical School and Hospital, then read a paper on "Tubercular hip and spine," which was a masterly presentation of this subject. Dr. Phelps illustrated his remarks with drawings and appliances for deformities original in character and instructive in a high degree. He received a round of applause as he finished his discourse. Dr. Phelps's paper was discussed by Drs. Roswell Park, Bernard Bartow, E. M. Dooley and Edward J. Meyer, and some interesting patients, illustrating several prominent features of the subject, were presented.

Then followed a paper on "Smallpox," by Professor William T. Corlett, of Cleveland, O., professor of dermatology in the Medical College of Western Reserve University. This subject was profusely illustrated with superb lantern-slide views, rare in character, and most difficult to obtain. Professor Corlett is an admirable speaker, understands his subject, and presents it in choice and forceful English. As he concluded he was paid the tribute of prolonged applause from an interested audience. This subject was discussed by Drs. A. W. Henchell, of Rochester, and Dr. E. C. W. O'Brien, of Buffalo. Dr. O'Brien was health officer of Buffalo in 1873 during a great epidemic of smallpox, when he saw upwards of 2,000 cases, and he gave some interesting experiences relating to that outbreak. It was at a time when all the larger cities were invaded, but in none was it so quickly brought under control and stamped out as in Buffalo.

The association then adjourned.

In the evening, at 6.30 o'clock, the annual dinner was served at the Hotel Iroquois, at which there were good viands, good speeches and good cheer.

And so ended a very interesting and enjoyable day, for the planning and carrying out of the numerous details of which the executive committee deserves the thanks of everyone who participated in the several events.

